# Obstetric risk factors contributing to postpartum depression in the Northern Region of Ghana

**DOI:** 10.1371/journal.pmen.0000353

**Published:** 2025-07-07

**Authors:** Yussif Hamdan Adam, Prosper Mbawuni, Abdul-Raheem Mohammed

**Affiliations:** 1 Department of Social and Behavioural Change, School of Public Health, University for Development Studies, Tamale, Ghana; 2 Northern Regional Health Directorate, Ghana Health Services, Tamale, Ghana; PLOS: Public Library of Science, UNITED KINGDOM OF GREAT BRITAIN AND NORTHERN IRELAND

## Abstract

Postpartum depression (PPD) is a specific mental disorder that has a negative impact on the well-being of the mother and baby. Although several studies have explored the prevalence of PPD, they are dominated largely from Western and other Middle-East and Asian countries. The literature on the prevalence of PPD and the obstetric and demographic factors that contribute to this disorder is limited in sub-Saharan Africa, specifically Ghana. Thus, the present study examined the prevalence rate of PPD in the Northern Region of Ghana and the obstetric and demographic factors contributing to the disorder. The study employed a cross-sectional survey design. Three hundred and twenty-one (321) postpartum mothers participated in the study by completing the Edinburgh Postnatal Depression Scale and other questionnaires on sociodemographic and obstetric variables. Binary and multiple logistic regression were used for the analysis. The results showed a prevalence rate of 10.9% for PPD. Family history of mental health disorder, experience of stress within the past year, chronic physical health conditions, planned pregnancy, complications during pregnancy, childbirth complications, traumatic birth experience, and history of miscarriages were positively associated with PPD. The regression results showed that, the experience of stress within the past year (AOR = 8.54 [95%CI (3.277,22.277)], p < 0.001), planned pregnancy (AOR = 0.19 [95%CI (0.076,0.477)], p < 0.001) and complications during pregnancy predicted PPD outcomes. (AOR = 2.814 [95%CI (1.120,7.072)], p = 0.028). These findings have implications for the early identification and management of PPD.

## Introduction

Postpartum depression (PPD) is a prevalent and serious mental health condition that affects mothers globally, typically manifesting within the first year after childbirth [1]. It is characterised by symptoms such as intense sadness, anxiety, fatigue, and impaired functioning [2,3]. PPD can significantly hinder a mother’s ability to care for her newborn, disrupt maternal-infant bonding, and strain family relationships [4]. Research has shown that untreated PPD can lead to long-term negative outcomes for both mothers and their children, including developmental delays, behavioural issues, and compromised emotional health in children [5,6]. Given these implications, understanding and mitigating factors contributing to PPD is essential to public health. Although several studies have been conducted on PPD worldwide (i.e., [7–10], most of these studies have been carried out in Western and other Middle-East and Asian countries as compared to sub-Saharan Africa.

PPD is a common mental health disorder affecting approximately 13% of women of childbearing age. The prevalence is higher among teenage mothers [11]. The World Health Organization [12] estimates that depression, including PPD, contributes substantially to the global burden of neurological and mental health disorders, ranking as the fourth leading cause of disability and premature death. PPD typically starts within the first six weeks after childbirth and often requires intervention from a healthcare professional. For some women, the initial “baby blues” persist and intensify, while others experience a period of stability after delivery, followed by a gradual onset of depressive symptoms [13].

Recent studies on the global prevalence of PPD reported rates of between 17.22% and 19.18% [14,15]. The prevalence of PPD is higher in the Middle-East and Asia when compared with Western countries [9]. Findings from a recent meta-analysis involving 412 studies of 792,055 women across 46 countries ranged from 3% in Singapore to a higher rate of 44% in South Africa [14]. Another meta-analysis with developing countries, including China, showed that China had a prevalence rate of 26.32% [8].

In Africa, PPD affects approximately one in ten women [16]. Some countries report higher prevalence rates, including Uganda at 43.0% and Cameroon at 23.4%, while Ethiopia and Morocco show lower rates at 13.1% and 11.6%, respectively. Socio-demographic factors—such as religion, age, socioeconomic status, education level, and unemployment—are associated with PPD [17]. Key risk factors consistently identified over time include a history of depression, lack of social support, poor relationship quality with a partner, and high stress levels [18].

In Ghana, some attempts were made to explore the prevalence rates among different groups, which produced different rates. A study by Daliri et al. [19] found a high prevalence rate of PPD at 50.4% in a conflict-prone community in Ghana. Another study involving a larger sample size in three different health facilities in Accra found a prevalence rate ranging from 8.6%, 31.1%, and 41.1%, respectively. A phenomenological study exploring the experience of mothers attending PPD consultations emerged themes that listed experiences such as anger, guilt and insomnia [20]. In a multinational study involving postpartum mothers visiting primary health centres in Egypt, Yemen, Iraq, Syria and Ghana, a prevalence rate of 13.6% on average was found, with Ghana having the highest prevalence rate of 26%. [7]. From a systematic review of ten studies from Ghana [21], a total of thirteen distinct prevalence estimates for PPD were extracted and subsequently compared. Whiles the study by Sefogah et al. [22] in the Greater Accra Region involving a larger sample of 1456 participants reported a higher prevalence rate of 27.5% the study by Anokye et al. [23] in the Ashanti region with 257 participants, reported a relatively lower PPD prevalence of 7%.

Previous studies have reported the relationship between obstetrics, sociodemographic factors and PPD. For instance, gestational diabetes mellitus, depression during pregnancy, giving birth to boys, and epidural anaesthesia during delivery were found to be significantly associated with PPD [8]. Zanardo et al. [24] showed no significant difference in PPD among Italian women who had vaginal and Caesarean Section. Another study among young postpartum mothers in the United Arab Emirates showed a prevalence rate of 35%, and it was also found that being employed on a part-time basis was associated with higher risks of developing PPD [25]. Among a sample of postpartum mothers in Kosovo, complications during pregnancy, fear of childbirth, prenatal depression or anxiety, and marital relations were the significant predictors of PPD [26]. Obstetric labour pain has also been found to be associated with PPD [27]. Positive childbirth experience has been found to be negatively associated with the development of PPD [28]. A prospective cohort study among pregnant women who experienced threatened preterm labour, compared with a control group, found a higher likelihood of PPD diagnosis [29]. Additionally, lower educational attainment, advanced maternal age, history of traumatic experience, and elevated trait anxiety were identified as major predictors of PPD [29].

### The present study

Despite the presence of literature on PPD globally, it is dominated by contributions from Western, Middle-East and Asian countries. There are limited studies on the prevalence of PPD in sub-Saharan Africa, including Ghana. Moreover, the role of obstetric and socio-demographic factors has received less attention in research. Hence, the present study seeks to contribute to the literature on PPD by exploring the prevalence rate of PPD in the Northern Region of Ghana and the contribution of obstetrics and sociodemographic factors to PPD.

## Method

### Study setting and sampling

The study was conducted at the Northern Regional Hospital in Tamale, Ghana. This is a tertiary-level health facility. Postpartum mothers at the facility’s postnatal clinic were chosen at random using the lottery approach. A total of 3134 mothers who delivered within the period at the hospital from the postnatal register were the targeted population, where numbers written on pieces of papers were assigned to represent each mother before arriving at the target sample Their antenatal cards were reviewed to ensure that each mother selected met the selection criteria for the participation in the study. The inclusion criteria were mothers who had live births, were within 2 – 8 weeks (14–56 days) after delivery, and were 18 years and above. Postpartum mothers who were seriously ill and those who had miscarriages or stillbirths were also excluded from the study.

### Participants

The sample size was determined a priori using the G-Power software. Estimating a power of 90%, two-tailed tests, and medium effect sizes, the minimum required sample size arrived at was 269. To account for potential incomplete data from some respondents, 321 postpartum mothers participated in the study. Table 1 summarizes the demographic characteristics of the study participants. The majority (46.7%) of the respondents were within 18–24 years, while 13.4% were within the age of 35–49 years. A large section (87.9%) of the respondents were married, and most (47.6%) of them were Christians. Regarding educational status, 41.4% had a tertiary qualification, while a few (3.8%) had no formal education. A large proportion of the mothers (58.3%) were traders. The study followed the ethical guidelines espoused by the Helsinki Declaration [30] and was approved by the Ghana Health Service Ethics Review Committee (with the approval number GHS-ERC 038/11/23). All participants provided written informed consent before completing the questionnaire. The data collection was carried out between 28th March 2024 and 29th May 2024.

**Table 1:** Demographic characteristics of the respondents.

Variables	Frequency (%)
**Age**	
18-24	150(46.7)
25-34	128(39.9)
35-49	43(13.4)
**Marital Status**	
Co-habitation	38(11.83)
Divorced	1(0.003)
Married	282(87.85)
**Religious Affiliation**	
Christian	153(47.7)
Muslim	152(47.4)
Traditionalist	16(5.0)
**Educational Level**	
No formal education	12(3.7)
Primary school	36(11.2)
Junior High School	54(16.8)
Senior High School	86(26.8)
Tertiary	133(41.4)
**Occupation**	
Civil Servant	88(27.4)
Farmer	15(4.7)
Housewife	27(8.4)
Trader/Businessperson	187(58.3)
Others	4(1.2)

### Research design and procedure

A cross-sectional study design was used for this study. The participants were presented with a set of questionnaires to complete at once. The questionnaire was administered in the English language except for a few instances where some mothers had to complete the questionnaire in the local language (Dagbani). In those instances, three Nurses who were proficient in both English and the local language assisted in the administration of the questionnaires. The questionnaire was made up of three sections. Section A collected data on the demographic characteristics of the participants, while section B focused on the obstetric factors (i.e., underlying medical conditions, history of births and physical condition). Section C assessed levels of depression using the Edinburgh Postnatal Depression Scale [31]. This scale has demonstrated good psychometric properties in the present study (Chronbach’s α = .90) and has also been validated in a previous study in Ghana [22]. This scale is made up of 10 items with 4 options presented in Likert-scale format. Responses to questions 1, 2, and 4 are ranked 0,1,2,3, depending on how severe the symptoms were. It is reverse-scored for questions 3 and 5–10. The whole score, which has a maximum score of 30, is calculated by summing up the scores for each of the 10 items. A mother who had a score of 10 or higher on the depression rating scale was considered to be depressed, as the study’s cutoff point was 10. All mothers completed the questionnaires in the morning since the postnatal clinic is open on weekdays in the mornings.

### Data analysis

The data was cleaned and entered into Microsoft Excel version 2021 and exported to SPSS version 20 for analysis of means, frequencies, and associations. To determine the obstetric risk factors (predictor variables) associated with PPD, Binary logistic regression was used to model the likelihood that an observation belongs to one of these two groups (dependent variable and the predictors). To shed light on the relative contributions of each predictor variable to the chance of PPD developing in women, the variables that showed a significance level (p < 0.05) during bivariate analysis (Model 1) were included in the multivariate regression analysis (Model 2). The findings from the bivariate analysis indicated that eight predictor factors were strongly linked to mothers’ PPD. To account for confounders, a multivariate logistic regression model was applied.

## Results

A greater percentage of the mothers (98.2%) gave birth through vaginal delivery, whereas 7.8% had a Caesarean procedure. A third of the mothers’ babies were aged 4 weeks or more (33.5%), followed by those aged 2 weeks (28.7%). Similarly, about a third of the mothers had three children (32.1%). A higher proportion (87.5%) of the mothers were categorised as multiparous. This is illustrated in Table 2.

**Table 2:** Summary statistics of the obstetric factors of the postpartum mothers.

Variables	Frequency (%) n = 321
**Mode of delivery**	
Caesarean Section	25(7.8)
Vaginal delivery	296(92.2)
**Pregnancy outcome**	
Preterm	6(1.9)
Term - Life birth	315(98.1)
**No. of Children**	
0	5(1.6)
1	53(16.5)
2	86(26.8)
3	103(32.1)
4	32(10.0)
5+	42(13.1)
**Category of Mothers**	
Multiparous	281(87.5)
Primiparous	40(12.5)
**Age of Baby in weeks**	
< 7days	25(7.8)
1 week	39(12.1)
2 weeks	92(28.7)
3 weeks	57(17.8)
4 weeks or more	108(33.5)

### Prevalence of PPD among mothers

**Fig 1:**
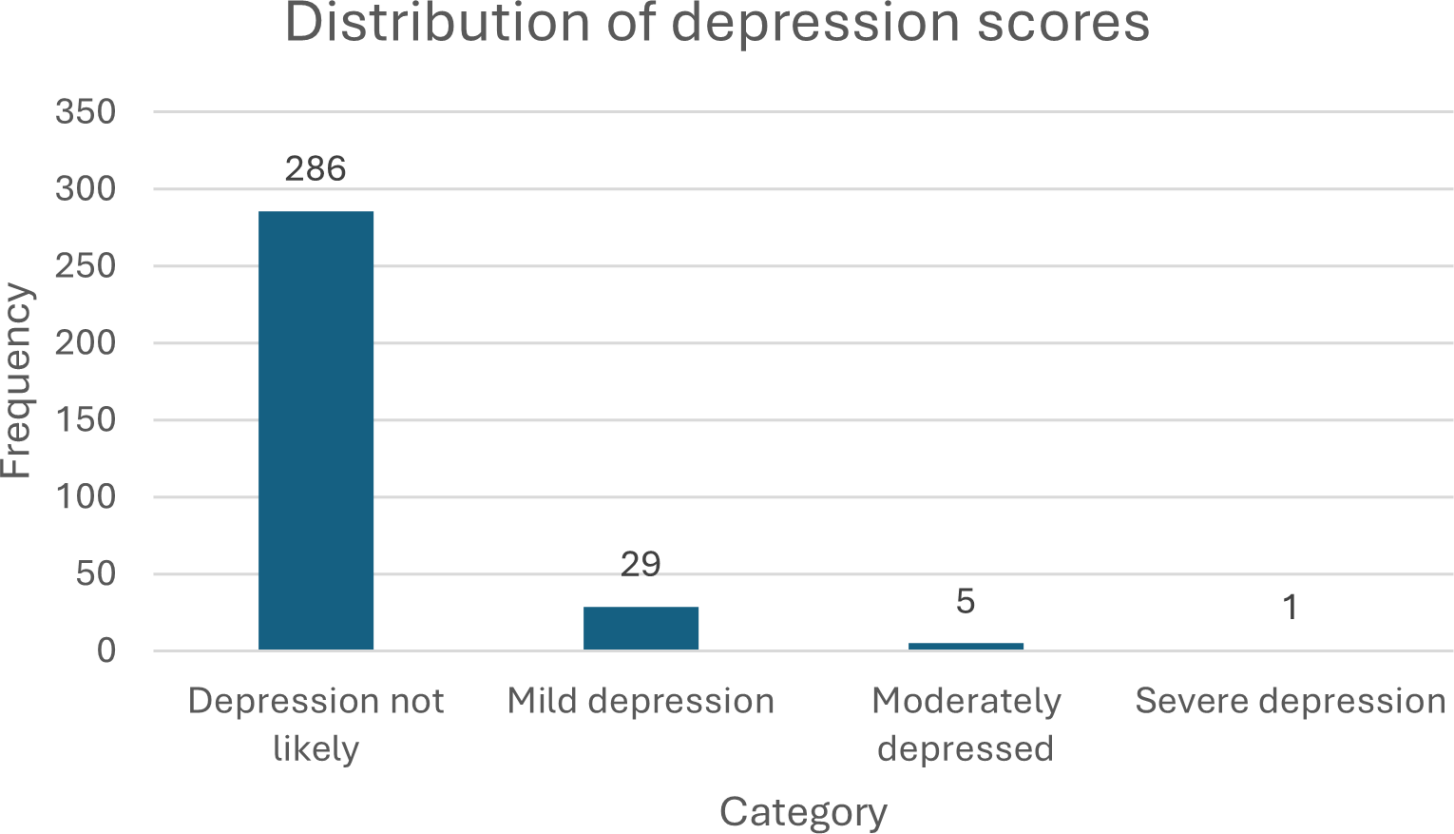
Level of depression among the postpartum mothers. The results of the binary logistic regression on the association between the demographic factors and PPD showed that while marital status χ^2^(3) = 5.9, *p* = .12, age χ^2^(2) = 0.73, *p* = .69, education χ^2^(4) = 6.45, *p* = .19, occupation χ^2^(4) = 5.89, *p* = .21, pregnancy outcome χ^2^(1) = 0.18, *p* = .67, and category of mothers χ^2^(1) = 1.81, *p* = .18 had no relationship with PPD, mode of delivery χ^2^(1) = 6.2, *p* = .013 and number of children χ^2^(5) = 14.8, *p* = .01 had a significant association with PPD.

The majority of the 321 mothers in the sample (89.1%) were found to be unlikely to be depressed based on the depression scores obtained, however, a significant number (10.9%) fell into the mild to severe depression categories. This is illustrated in Fig 1.

The relationship between obstetric variables and PPD showed varied outcomes, as summarised in Table 3. On personal and family history, specific factors such as Family history of mental health disorder, significant life stressors in the past, and chronic physical health conditions were associated with PPD whereas obstetric factors such as planned pregnancy, complications during pregnancy, complications during childbirth, traumatic birth experience, and history of miscarriage or stillbirth were associated with PPD. The results of multivariate regression analysis showed that significant life stressors in the past were nearly nine times more likely to be depressed than their peers who did not experience any life stressors (AOR = 8.54 [95%CI (3.277,22.277)], p < 0.001). Pregnancy-related psychological disorders were less common among respondents who planned their pregnancy than in respondents who did not (AOR = 0.19 [95%CI (0.076,0.477)], p < 0.001). Additionally, compared to respondents who had not encountered any pregnancy-related difficulties, those who had encountered the difficulties were about three times more likely to suffer depression (AOR = 2.814 [95%CI (1.120,7.072)], p = 0.028).

**Table 3:** Bivariate and multivariate analysis of risk factors associated with postpartum depression.

Risk Factors		Postpartum Depression		COR (95%CI)	p-value	AOR (95%CI)	p-value
		No	Yes	Model 1		Model 2	
**Personal and Family History**							
Previous history of mental health issues	No	234(81.8)	24(68.6)	1 (Ref)		1 (Ref)	
	Yes	52(18.2)	11(31.4)	2.062(0.951,4.474)	0.067	0.851(.342,2.119)	0.729
Immediate family diagnosed or experienced depression or any other mental health disorder	No	244(85.3)	18(51.4)	1 (Ref)		1 (Ref)	
	Yes	42(14.7)	17(48.6)	5.487(2.62,11.492)	**<.001**	1.779(.724,4.375)	0.209
Experienced any significant life stressors in the past year	No	246(86.0)	12(34.3)	1 (Ref)		1 (Ref)	
	Yes	40(14.0)	23(65.7)	11.787(5.437,25.556)	**<.001**	8.544(3.277,22.277)	**<.001**
History of chronic physical health conditions	No	280(97.9)	30(85.7)	1 (Ref)		1 (Ref)	
	Yes	6(2.1)	5(14.3)	7.778(2.239,27.015)	**<.001**	1.832(.478,7.019)	0.377
**Pregnancy and Childbirth factors**							
Planned pregnancy	No	84(29.4)	27(77.1)	1 (Ref)		1 (Ref)	
	Yes	202(70.6)	8(22.9)	0.123(.054,0.282)	**<.001**	0.19(0.076,0.477)	**<.001**
Experience any complications during pregnancy	No	255(89.2)	18(51.4)	1 (Ref)		1 (Ref)	
	Yes	31(10.8)	17(48.6)	7.769(3.631,16.620)	**<.001**	2.814(1.120,7.072)	**0.028**
Any complications during childbirth?]	No	277(96.9)	28(80.0)	1 (Ref)		1 (Ref)	
	Yes	9(3.1)	7(20.0)	7.694(2.662,22.241)	**<.001**	3.828(0.794,18.444)	0.094
Traumatic birth experience?]	No	278(97.2)	29(82.9)	1 (Ref)		1 (Ref)	
	Yes	8(2.8)	6(17.1)	7.190(2.333,22.156)	**<.001**	1.516(0.234,9.845)	0.663
History of miscarriage or stillbirth?]	No	266(93.0)	25(71.4)	1 (Ref)		1 (Ref)	
	Yes	20(7.0)	10(28.6)	5.320(2.245,12.606)	**<.001**	2.023(0.730,5.604)	0.175

Note: COR = crude odds ratio, AOR = adjusted odds ratio, CI = confidence interval.

## Discussion

The present study explored the prevalence of PPD among mothers and the contribution of obstetric and socio-demographic factors to PPD. The overall prevalence of PPD, considering all levels (mild, moderate, and severe), was 10.9 percent. This suggests that a significant proportion of mothers in the sample experienced some degree of possible depression, which aligns with existing research indicating that PPD is relatively common among new mothers. This is consistent with previous studies in Ghana reporting similar rates [22,23]. Most mothers (89.1%) scored below 9 on the EPDS, indicating that depression is not likely. However, a proportion of the mothers (9.0%) scored between 10 and 14, indicating mild depression. A smaller percentage of mothers (1.6%) scored between 15 and 20, indicating moderate depression. Only one mother (0.3%) scored 21 and above, indicating severe depression. However, these rates are inconsistent with previous studies, such as Daliri et al. [19], which reported a higher prevalence rate of 50.4% in a conflict-zoned community in Ghana. The distribution of PPD highlights the spectrum of PPD severity among the surveyed mothers, with the majority experiencing either no depression or mild symptoms. It is important to note that even mild depression can have a significant impact on a mother’s well-being, which requires some level of attention and support.

The results further showed an association and contribution of some demographic factors to PPD. Key among these factors were mode of delivery and number of children. This meant that mothers who gave birth through Caesarean section were more likely to develop PPD than the vaginal birth delivery. This is consistent with several studies, including longitudinal studies and meta-analysis [32,33]. However, a previous study found no significant association between mode of delivery and PPD [34]. Moreover, we found that mothers’ number of children had a significant impact on PPD. This meant that mothers who had given birth for the first time or had fewer children were more likely to develop PPD than those with more children. This is consistent with previous studies showing that first-time mothers had a higher chance of developing PPD than those with children [35–37]. This suggests that after multiple successful births, it could serve as a protective factor against the development of PPD.

The study revealed a trend in the potential contribution of obstetric factors to PPD. While mothers with a history of mental health illness had an increased risk of PPD, this association was not statistically significant after adjusting for other factors. Mothers with family members diagnosed with mental health disorders showed significantly higher odds of experiencing PPD, indicating a potential familial predisposition to PPD. While mothers with chronic physical health conditions showed an increased risk of PPD in the unadjusted model, this association was not significant after adjusting for other variables. Mothers with planned pregnancies had a significantly lower risk of PPD, suggesting that planned pregnancy served as a protective factor against PPD. Mothers who experienced complications during pregnancy had a higher risk of PPD, emphasising the role of pregnancy-related health issues in PPD. The findings of this study corroborate with other studies, which found a significant correlation between PPD and several obstetric risk factors [27,38–41].

The recent WHO situational analysis on PPD in Ghana reveals several critical insights into the state of maternal mental health in the country [42]. According to the analysis, mental health disorders, including PPD, have a significant impact on both maternal well-being and infant health, with strong links to poor infant growth and social adversity. However, despite this evidence, there is a lack of sufficient provision in the healthcare system for screening, early detection, and effective management of maternal mental health issues. Hence, the results of the current study could help in improving the policy guidelines on the prevention and management of PPD in Ghana.

The present findings have important implications for recognising and addressing PPD as a major threat to public health. Understanding the prevalence and severity of PPD allows healthcare providers and support systems to better assist in the mental health and well-being of new mothers throughout the essential postpartum period by implementing appropriate interventions. While the prevalence of severe PPD is low in this study, healthcare providers should still give attention to early screening and addressing depression among new mothers. Provision of routine screening, during antenatal and postpartum care visits, staff should conduct routine PPD checks using validated instruments like the EPDS. For mothers who are experiencing or at risk of depression, this will make early detection and intervention easier. This means that the staff offering health services to the pregnant and postpartum mothers will require special training and workshops integrating the factors predicting PPD as found in the present study. Health facilities should, as part of measures to mitigate the impact of PPD, offer comprehensive support services tailored to the needs of mothers experiencing PPD, including access to counselling, support groups, and psychiatric care. Programmes for postnatal care should incorporate these services, and all mothers should have easy access to them. Health staff (Midwives) should provide psychoeducation to mothers and their families about PPD, its symptoms, risk factors, and available support systems. This will help increase awareness and promote early help-seeking behaviour among affected individuals. Advocacy for policies that prioritise maternal mental health and allocate resources for PPD prevention, screening, and treatment programmes. Policy support is essential for creating supportive environments and systems that promote maternal well-being and early intervention for PPD.

The focus on live births and the exclusion of stillbirths at term or preterm are considered limitations of the study. Considering that stillbirths are significant stressors, they could have the potential to cause the development of PPD, thereby affecting the prevalence rate. Hence, future studies should consider including mothers who had stillbirths among the study sample. The EPDS used to measure PPD generally requires participants to reflect within the past seven days on what they felt about each item. Due to the limitations of cross-sectional surveys, their experiences might vary depending on the week or time of assessment. Thus, future studies should consider an experience sampling methodology that will involve measuring PPD continuously for several weeks right from birth, which will increase the validity of the present findings.
